# Investigating the impact of financial concerns on symptoms of depression in UK healthcare workers: data from the UK-REACH nationwide cohort study

**DOI:** 10.1192/bjo.2023.520

**Published:** 2023-07-12

**Authors:** Martin McBride, Christopher A. Martin, Lucy Teece, Patricia Irizar, Megan Batson, Susie Lagrata, Padmasayee Papineni, Joshua Nazareth, Daniel Pan, Alison Leary, Katherine Woolf, Manish Pareek, the UK-REACH Study Collaborative Group

**Affiliations:** Department of Psychiatry, Leicester Partnership NHS Trust, UK; Department of Respiratory Sciences, University of Leicester, UK; and Department of Infection and HIV Medicine, University Hospitals of Leicester NHS Trust, UK; Biostatistics Research Group, Department of Population Health Sciences, University of Leicester, UK; Department of Sociology, School of Social Sciences, Faculty of Humanities, University of Manchester, UK; Leicester Medical School, University of Leicester, UK; Queen Square Institute of Neurology and National Hospital for Neurology and Neurosurgery, University College London Hospitals NHS Foundation Trust, UK; Department of Infectious Diseases, Ealing Hospital, London North West University Healthcare NHS Trust, UK; Department of Respiratory Sciences, University of Leicester, UK; Department of Infection and HIV Medicine, University Hospitals of Leicester NHS Trust, UK; and Li Ka Shing Institute for Health Information and Discovery, Oxford Big Data Institute, UK; Department of Health and Social Care, London South Bank University, UK; UCL Medical School, University College London, UK

**Keywords:** Depressive disorders, health economics, epidemiology, psychiatric nursing, aetiology

## Abstract

**Background:**

Exploration of the association between financial concerns and depression in UK healthcare workers (HCWs) is paramount given the current ‘cost of living crisis’, ongoing strike action and recruitment/retention problems in the National Health Service.

**Aims:**

To assess the impact of financial concerns on the risk of depression in HCWs, how these concerns have changed over time and what factors might predict financial concerns.

**Method:**

We used longitudinal survey data from a UK-wide cohort of HCWs to determine whether financial concerns at baseline (December 2020 to March 2021) were associated with depression (measured with the Public Health Questionnaire-2) at follow-up (June to October 2022). We used logistic regression to examine the association between financial concerns and depression, and ordinal logistic regression to establish predictors of developing financial concerns.

**Results:**

A total of 3521 HCWs were included. Those concerned about their financial situation at baseline had higher odds of developing depressive symptoms at follow-up. Financial concerns increased in 43.8% of HCWs and decreased in 9%. Those in nursing, midwifery and other nursing roles had over twice the odds of developing financial concerns compared with those in medical roles.

**Conclusions:**

Financial concerns are increasing in prevalence and predict the later development of depressive symptoms in UK HCWs. Those in nursing, midwifery and other allied nursing roles may have been disproportionately affected. Our results are concerning given the potential effects on sickness absence and staff retention. Policy makers should act to alleviate financial concerns to reduce the impact this may have on a discontent workforce plagued by understaffing.

Depression is highly prevalent in healthcare workers (HCWs). A recent meta-review including over 3.2 million HCWs determined that, since the COVID-19 pandemic, the prevalence of depression in HCWs is 14–37% globally^1^. Here, nurses were highlighted as having the highest prevalence of depressive symptoms compared with other HCW roles. The rate of suicide in nurses has been estimated to be 23% higher than the national average in the UK.^2^ Gilleen et al^3^ found that the prevalence of low mood, anxiety and stress symptoms in HCWs increased during the pandemic when compared with pre-pandemic measures, although recent evidence suggests that these changes may not be sustained in the general population.^4^ Whether this increased prevalence will be sustained is yet to be determined, but any mechanisms that underlie such increases are important to recognise and address.

Depression incurs a significant personal cost to HCWs. In addition, healthcare organisations incur large financial costs as a result of mental health problems in their employees. The sickness absence rate of nurses, midwives and ambulance staff is roughly three to four times the national average for the labour force, and 20–30% of sick days in the UK National Health Service (NHS) are because of mental health problems.^5,6^

## Financial concerns and their association with depression

It is well established that low household income is associated with an increased risk of mental health problems.^7^ The UK's Office for National Statistics (ONS) has highlighted the difference in the prevalence of depression in those who believe they have the ability ‘to afford an unexpected expense’ compared with those who do not.^8^ Evidence concerning the relationship between financial concerns and depression in HCWs is limited and comes from small studies with cross-sectional designs. An Italian study from 2020 reported a non-significant increased risk of depression for HCWs with income loss as a result of COVID-19.^9^ A previous study in a HCW population from Pakistan demonstrated an association between financial concerns and depression.^10^ A recent study from Afghanistan found HCWs who reported a low monthly income to be at higher risk of depression than those in higher wage brackets.^11^

## Study aims

To date, there has been no longitudinal exploration of how financial concerns have affected the mental health of HCWs. This is a highly topical issue given the ongoing pay disputes and strike action by HCW groups in the UK.^12^ We therefore conducted an analysis of data collected between December 2020 and October 2022 from the nationwide UK Research Study into Ethnicity and COVID-19 Outcomes in Healthcare Workers (UK-REACH) longitudinal cohort study.^13^ This was a secondary analysis, the primary aim of which was to determine if financial concerns at baseline were associated with development of symptoms of depression at follow-up. Secondary aims were to determine if the prevalence of financial concerns among HCWs has changed over the course of the study, and to determine whether there are demographic or occupational predictors of developing financial concerns in HCWs.

## Method

### Overview

UK-REACH is a research programme that was established to investigate the disproportionate impact of the COVID-19 pandemic on HCWs from ethnic minority groups. In this work, we use data from the baseline questionnaire (administered December 2020 to March 2021) and the wave 4 questionnaire (administered June to October 2022, hereafter referred to as the ‘follow-up questionnaire’) of the prospective nationwide cohort study. We elected to examine the earliest and latest time points that we had data for because we expected that it would take considerable time for the effects of financial concerns to affect the mental health of participants. Furthermore, we wished to capture the effects of the UK's cost of living crisis on the prevalence of financial concerns. For a detailed overview of the study, see the study protocol^13^ and cohort papers.^14^ All measures collected in the questionnaires are available in the data dictionary (https://www.uk-reach.org/data-dictionary).

#### Ethical approval

UK-REACH was approved by the Health Research Authority (Brighton and Sussex Research Ethics Committee; ethics reference number 20/HRA/4718). All participants gave informed written (electronic) consent. The UK-REACH study is registered at ISRCTN (reference: ISRCTN 11811602).

#### Involvement and engagement

We worked closely with a Professional Expert Panel composed of an ethnically and occupationally diverse group of HCWs, as well as with national and local organisations, to help shape the research question and analysis plan.^13^ Two of the panel (S.L. and P.P.) are co-authors.

### Study population

To be recruited into the cohort study participants had to be living in the UK, currently employed as a HCW or ancillary worker in a healthcare setting and/or registered with a participating UK healthcare regulatory body, and aged ≥16 years.^13^

### Recruitment

Recruitment into the study has been described in previous work.^14–19^ In brief, participating healthcare regulators (for a list see the supplementary material available at https://doi.org/10.1192/bjo.2023.520) emailed their registrants to invite them to participate in the study. A small proportion of the cohort were recruited directly by hospital Trusts and advertising in newsletters/social media.^14^ Those interested in participating accessed the study website, where they could provide informed electronic consent and complete the baseline questionnaire. Invitations to complete subsequent questionnaires were emailed to participants who provided consent.

### Outcome measures and covariates

#### Screening for depression

Our primary outcome measure was meeting the screening threshold for depression on the Patient Health Questionnaire-2 (PHQ-2)^20^ at follow-up. The PHQ-2 comprises two questions concerning the frequency of low mood and anhedonia over the preceding 2 weeks, with each question being scored on a four-point scale from 0 (‘not at all’) through to 3 (‘nearly every day’). The sum of the scores from each question are combined to provide a single score (on a scale from 0 to 6). In the primary analysis, we use the validated cut-off point of ≥3 to indicate meeting screening criteria for depression.^20^ In a sensitivity analysis, we use the combined score as a continuous outcome measure.

#### Future financial concerns

Participating HCWs were asked the following question in both the baseline and the follow-up questionnaire, ‘How worried are you about your future financial situation?’, derived from questionnaire material developed as part of the Wellcome Trust's Longitudinal Population Studies COVID-19 questionnaire.^21^ This was supported by the Wellcome ‘Longitudinal Population Study COVID-19 Steering Group and Secretariat’ as a Strategic Support Science Grant (identifier 221574/Z/20/Z). Answers were on a five-point scale (1, ‘not at all’; 2, ‘a little bit’; 3, ‘moderately’; 4, ‘quite a bit’; 5, ‘extremely’). The measure collected at baseline was used to address the primary aim of the study (i.e. determining whether financial concerns predict the development of depression symptoms), and the measure collected in the follow-up questionnaire was used as an outcome measure in both secondary analyses (i.e. determining whether the prevalence of financial concerns has changed over the course of the study and whether there are particular occupational and demographic groups at risk of financial concerns).

#### Covariates

We adjusted for the following covariates in multivariable analyses: (a) demographic characteristics (age, sex assigned at birth, ethnicity), with ethnicity categorised according to the five broad ONS categories (White, Asian, Black, mixed, other);^22^ (b) occupation, collapsed into five categories (‘medical’, ‘nurses, nursing associates, midwives’, ‘allied health professionals and those in pharmacy, clinical sciences and optical roles’, ‘dental’ and ‘administrative/estates/other’^16,17,19^) and (c) deprivation in residential area, as determined by the Index of Multiple Deprivation (IMD) quintile.^24^

These covariates are derived from information provided in the baseline questionnaire. We examine the association of the same variables in the analysis of occupational and demographic predictors of financial concerns at follow-up.

### Statistical analysis

We excluded those with missing data for the primary outcome of interest (PHQ-2 at wave 4) and the primary exposure of interest (financial concerns at baseline), including those who answered ‘prefer not to answer’ to the relevant question. For the secondary analyses, we additionally excluded those with missing data on financial concerns at follow-up.

We summarised categorical variables with frequency and percentages, and non-normally distributed continuous variables with median and interquartile range. We used univariable and multivariable logistic regression to determine unadjusted and adjusted associations of financial concerns at baseline with meeting screening criteria for depression at follow-up. Multivariable analyses were adjusted for the all covariates above, as well as the PHQ-2 score at baseline. We presented the results as odds ratios or adjusted odds ratios (aORs), with 95% confidence intervals.

To investigate changes in financial concerns between the baseline and follow-up questionnaires, we examined a contingency table of these variables and tested whether the proportion in each outcome category remained consistent across the two time points, using a marginal homogeneity (Stuart–Maxwell) test.

To determine demographic and occupational predictors of financial concerns at follow-up, we used univariable and multivariable ordinal logistic regression. We tested the proportional odds assumption with the Brant test. Any violations were investigated by deriving binary variables for each cut-off point in the categorical outcome (i.e. not at all versus at least a little bit, not at all or a little bit versus at least moderately, etc.). We then fit logistic regression models with these as outcomes and plotted results to determine which variables violate the assumption.

We conducted two sensitivity analyses. In the first, we used univariable and multivariable linear regression (adjusted for the same covariates) to investigate whether examining the PHQ-2 score as a continuous outcome measure (rather than a binary measure) had any effect on the associations found in the logistic regression models used for our primary analysis. In the second, to investigate bias resulting from loss to follow-up, we compared the measures collected in the baseline questionnaire and used in the main analysis (sociodemographic, occupational, financial concerns and PHQ-2 score) in the cohort who did not respond to the follow-up questionnaire with those that did respond.

Multiple imputation by chained equations was used to impute missing covariate data in all models. The imputation model contained all variables except the one being imputed, including the outcome measure. Rubin's Rules were used to combine parameter estimates and standard errors from ten imputations into a single set of results.^23^ Although indices of deprivation are available for UK countries outside England, these are not directly comparable with the English IMD.^24^ We therefore elected to code IMD data as missing for those outside England and impute the missing information.

All analyses were conducted with Stata version 17 for macOS (StataCorp, College Station, Texas, USA) Figures were drawn with GraphPad Prism (version 9.4.0 for macOS; GraphPad Software, San Diego, California, USA; www.graphpad.com).

## Results

### Formation and description of the analysed sample

Recruitment into the study and formation of the analysed sample is detailed in Supplementary Figure 1. A total of 15 199 HCWs provided a response to the baseline questionnaire. Of these, 3891 responded to the follow-up; 330 were excluded for not providing information on baseline or follow-up PHQ-2 items and/or the baseline financial concerns item, meaning that 3521 HCWs were included in the primary analysis (23.2% of those who responded to the baseline questionnaire). A further three HCWs did not provide information on their level of financial concerns at follow-up, therefore 3518 were included in the secondary analyses.

Median age was 48 (interquartile range 37–56) years, 74.8% were female and 22.7% were from ethnic minority groups. A total of 819 (23.3%) were employed in medical roles; 756 (21.5%) were registered nurses, midwives and healthcare assistants; and 1471 (41.8%) were allied health professionals or working in pharmacy, clinical sciences or optical roles. At baseline, 12.1% met screening criteria for depression and 56% were at least a little concerned about their future financial situation (Table 1).

**Table 1:** Description of the analysed cohort (*N* = 3521)

Variable	Description
Age in years, median (IQR)	48 (37–56)
Missing	11 (0.3)
Sex assigned at birth	
Male	881 (25.0)
Female	2635 (74.8)
Missing	5 (0.1)
Ethnicity	
White	2645 (75.4)
Asian	536 (15.2)
Black	91 (2.6)
Mixed	129 (3.7)
Other	45 (1.3)
Missing	66 (1.9)
Occupation	
Medical	819 (23.3)
Nursing	756 (21.5)
Allied health professionals	1471 (41.8)
Dental	196 (5.6)
Admin/estates/other	183 (5.2)
Missing	96 (2.7)
Index of Multiple Deprivation quintile	
1 (most deprived)	279 (7.9)
2	479 (13.6)
3	634 (18.0)
4	771 (21.9)
5 (least deprived)	941 (26.7)
Missing	417 (11.8)
Depression (PHQ-2) score at baseline	
<3	3096 (87.9)
≥3	425 (12.1)
Degree of concern about future financial situation at baseline	
Not at all	1550 (44.0)
A little	1451 (41.2)
Moderately	312 (8.9)
Quite a bit	140 (4.0)
Extremely	68 (1.9)

All data are *n* (%) unless otherwise stated. The nursing category includes healthcare assistants, nursing associates and midwives. The allied health professionals category includes healthcare scientists, ambulance workers, pharmacists and those in optical roles. IQR, interquartile range; PHQ-2, Patient Health Questionnaire-2.

### Association of financial concerns and developing symptoms of depression

A description of the cohort stratified by the outcome measure (meeting screening criteria for depression at follow-up) together with unadjusted odds ratios for the association of each covariate with the outcome is shown in Table 2.

**Table 2:** Univariable analysis of the association between financial concerns and demographic/occupational factors at baseline with meeting depression criteria at follow-up

Variable	Follow-up PHQ-2 < 3 (negative depression screen), *n* = 3175 (90.2%)	Follow-up PHQ-2 ≥ 3 (positive depression screen), *n* = 346 (9.8%)	Odds ratio (95% CI)	*P*-value
Degree of concern about future financial situation				
Not at all	1461 (46.0)	89 (25.7)	Reference	–
A little	1304 (41.1)	147 (42.5)	1.85 (1.41–2.43)	<0.001
Moderately	264 (8.3)	48 (13.9)	2.98 (2.05–4.34)	<0.001
Quite a bit	100 (3.2)	40 (11.6)	6.57 (4.29–10.04)	<0.001
Extremely	46 (1.5)	22 (6.4)	7.85 (4.52–13.60)	<0.001
Age in years, median (IQR)	48 (38–56)	43 (34–52)	0.72 (0.65–0.79)	<0.001
Sex assigned at birth				
Male	805 (25.4)	76 (22.0)	Reference	–
Female	2365 (74.6)	270 (78.0)	1.21 (0.93–1.58)	0.16
Ethnicity				
White	2387 (76.7)	267 (78.3)	Reference	–
Asian	487 (15.6)	49 (14.4)	0.89 (0.64–1.22)	0.46
Black	83 (2.7)	8 (2.4)	0.85 (0.41–1.78)	0.68
Mixed	117 (3.8)	12 (3.5)	0.90 (0.49–1.65)	0.74
Other	40 (1.3)	5 (1.5)	1.12 (0.44–2.86)	0.82
Occupation				
Medical	773 (25.0)	46 (13.8)	Reference	–
Nursing	662 (21.4)	94 (28.1)	2.28 (1.59–3.28)	<0.001
Allied health professionals	1329 (43.0)	142 (42.5)	1.72 (1.23–2.42)	0.002
Dental	170 (5.5)	26 (7.8)	2.48 (1.51–4.09)	<0.001
Admin/estates/other	157 (5.1)	26 (7.8)	2.66 (1.60–4.43)	<0.001
Index of Multiple Deprivation quintile				
1 (most deprived)	230 (8.2)	49 (16.1)	1.69 (1.14–2.53)	0.01
2	418 (14.9)	61 (20.0)	1.19 (0.83–1.71)	0.34
3	564 (20.2)	70 (23.0)	Reference	–
4	713 (25.5)	58 (19.0)	0.65 (0.45–0.93)	0.02
5 (least deprived)	874 (31.2)	67 (22.0)	0.61 (0.44–0.86)	0.004
Depression (PHQ-2 score) at baseline				
<3	2905 (91.5)	191 (55.2)	Reference	–
≥3	270 (8.5)	155 (44.8)	8.7 (6.8–11.2)	<0.001

All data in the first two columns are *n* (%) unless stated otherwise; percentages are calculated column-wise, apart from the total number of those not meeting or meeting depression screening criteria, which are calculated row-wise. Odds ratios are from univariable logistic regression on the imputed data-set. Odds ratio for age is per decade increase. The nursing category includes healthcare assistants, nursing associates and midwives. The allied health professionals category includes healthcare scientists, ambulance workers, pharmacists and those in optical roles. PHQ-2, Patient Health Questionnaire-2; IQR, interquartile range.

The degree of participants’ concern about their future financial situation was strongly associated with meeting screening criteria for depression at follow-up. Those who were extremely concerned about finances at baseline had over seven times the odds of meeting the screening criteria for depression at follow-up compared with those who were not at all concerned at baseline (odds ratio 7.85, 95% CI 4.52–13.60).

**Fig. 1:**
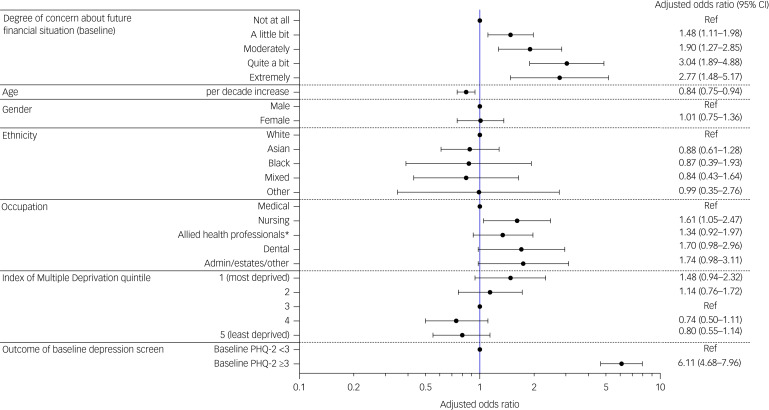
Multivariable logistic regression demonstrating the relationship between financial concerns at baseline and meeting depression screening criteria at follow-up, after adjustment for demographics, occupation and baseline depression screening outcome. PHQ-2, Patient Health Questionnaire-2; Ref, reference group for categorical variable. *Included in the 'Allied health professionals' group are healthcare scientists, pharmacists, ambulance workers and those in optical roles. Fig. 1 details the result of a mutivariable logistic regression analysis. Results are displayed as adjusted odds ratios (circles) and 95% confidence intervals (bars). Circles without bars are shown for the reference group of a categorical variable. Odds ratios are mutually adjusted for all variables in the figure.

After adjustment for age, sex assigned at birth, ethnicity, occupation, IMD quintile and baseline depression screening outcome (Fig. 1), greater future financial concerns at baseline were associated with higher odds of meeting screening criteria for depression (compared with not at all; a little: aOR 1.48, 95% CI 1.11–1.98; moderately: aOR 1.90, 95% CI 1.27–2.85; quite a bit: aOR 3.04, 95% CI 1.89–4.88; extremely: aOR 2.77, 95% CI 1.48–5.17). Increasing age was associated with lower odds of meeting screening criteria for depression at follow-up (aOR 0.84, 95% CI 0.75–0.94; per decade increase). Working in a nursing role compared with a medical role (aOR 1.61, 95% CI 1.05–2.47) was associated with higher odds of meeting screening criteria for depression at follow-up (see Fig. 1).

Sensitivity analysis of PHQ-2 score as a continuous outcome using linear regression did not materially change the findings (see Supplementary Table 1).

### Changes in financial concerns over time

A cross-tabulation of financial concerns at both time points is shown in Table 3: 44% reported no financial concerns at baseline compared with 22% at follow-up, 47.1% of the cohort reported the same level of financial concern at both time points, 43.8% reported an increased level of financial concern and 9% reported a decreased level of financial concerns at follow-up compared with baseline. The marginal homogeneity test was significant (*P* < 0.001), suggesting significant change in the proportion of HCWs concerned about their future financial situation between the two time points.

**Table 3:** Changes in the proportion of those with financial concerns between baseline and follow-up questionnaires

		Degree of financial concern at baseline				
		Not at all	A little bit	Moderately	Quite a bit	Extremely
Degree of financial concern at follow-up	Not at all	655 (42.3)	110 (7.6)	7 (2.2)	5 (3.6)	1 (1.5)
	A little bit	722 (46.6)	820 (56.6)	117 (37.5)	22 (15.7)	5 (7.4)
	Moderately	120 (7.8)	337 (23.2)	90 (28.9)	25 (17.9)	10 (14.7)
	Quite a bit	37 (2.4)	135 (9.3)	73 (23.4)	57 (40.7)	15 (22.1)
	Extremely	15 (1.0)	48 (3.3)	25 (8.0)	31 (22.1)	37 (54.4)

0–10%						
10–20%						
20–30%						
30–40%						
40–50%						
>50%						

The table shows a cross-tabulation of the cohort by their responses to the financial concerns question at baseline (columns) and at follow-up (rows). All data are *n* (column %). Marginal homogeneity (Stuart–Maxwell) test *P* < 0.001.

### Predictors of financial concerns at follow-up

Results of the univariable and multivariable ordinal logistic regression analyses to examine predictors of financial concerns at follow-up are shown in Table 4. After adjustment for age, sex assigned at birth, ethnicity, occupation, IMD quintile and baseline level of financial concerns, older HCWs had lower odds of having financial concerns (aOR 0.77, 95% CI 0.72–0.81). Those working in nursing or allied health professional roles (compared with medical roles) had higher odds of financial concerns at follow-up (nursing: aOR 2.28, 95% CI 1.84–2.82; allied health professional: aOR 1.64, 95% CI 1.37–1.97).

**Table 4:** Ordered logistic regression model demonstrating the univariable and multivariable association of demographic and occupational factors with increasing financial concerns at follow-up

Variable	Unadjusted odds ratio (95% CI)	*P*-value	Adjusted odds ratio (95% CI)	*P*-value
Age	0.73 (0.69–0.77)	<0.001	0.77 (0.72–0.81)	<0.001
Sex assigned at birth				
Male	Reference	–	Reference	−
Female	1.17 (1.01–1.35)	0.03	0.98 (0.84–1.14)	0.77
Ethnicity				
White	Reference	–	Reference	–
Asian	1.02 (0.86–1.22)	0.80	1.08 (0.90–1.31)	0.41
Black	0.97 (0.65–1.44)	0.88	0.79 (0.52–1.20)	0.27
Mixed	0.81 (0.58–1.13)	0.21	0.81 (0.57–1.15)	0.24
Other	0.93 (0.52–1.66)	0.80	0.96 (0.54–1.70)	0.89
Occupation				
Medical	Reference	–	Reference	–
Nursing	2.47 (2.06–2.98)	<0.001	2.28 (1.84–2.82)	<0.001
Allied health professionals	1.95 (1.66–2.29)	<0.001	1.64 (1.37–1.97)	<0.001
Dental	2.50 (1.86–3.37)	<0.001	1.48 (1.09–2.02)	0.01
Admin/estates/other	2.02 (1.49–2.73)	<0.001	1.41 (1.02–1.94)	0.04
Index of Multiple Deprivation quintile				
1 (most deprived)	1.30 (1.01–1.68)	0.04	1.08 (0.82–1.42)	0.01
2	1.12 (0.90–1.38)	0.31	1.02 (0.82–1.27)	0.34
3	Reference	–	Reference	–
4	0.71 (0.58–0.86)	<0.001	0.81 (0.67–0.99)	0.04
5 (least deprived)	0.66 (0.55–0.80)	<0.001	0.81 (0.67–0.98)	0.03
Degree of concern about future financial situation at baseline				
Not at all	Reference	–	Reference	–
A little	6.18 (5.28–7.23)	<0.001	5.62 (4.80–6.59)	<0.001
Moderately	16.65 (13.03–21.26)	<0.001	15.50 (12.10–19.85)	<0.001
Quite a bit	53.09 (37.65–74.86)	<0.001	44.71 (31.51–63.44)	<0.001
Extremely	172.31 (103.5–286.78)	<0.001	166.09 (99.02–278.58)	<0.001

The table shows univariable and multivariable ordered logistic regression with an outcome of financial concerns at follow-up (five-level ordinal variable). Parallel odds assumption was checked with the Brant test. This was significant at *P* < 0.001. We plotted odds ratios derived from logistic regression for each level of the outcome variable and determined that the parallel odds assumption was likely violated by inclusion of baseline score. We tested this hypothesis by removing baseline score from the model and performing the Brant test again, which returned a non-significant *P*-value (see Supplementary Appendix 1). Given the importance of adjusting for baseline score, but the lack of importance placed upon the relationship of this variable with the outcome, we elected to continue with the planned analysis of using ordered logistic regression and adjusting for baseline score (accepting that the odds ratios for baseline score may not be accurate for all levels of the outcome variable). Odds ratio for age is per decade increase. The nursing category includes healthcare assistants, nursing associates and midwives. The allied health professionals category includes healthcare scientists, ambulance workers, pharmacists and those in optical roles.

For details of the post-estimation analysis of the validity of using ordinal logistic regression for this analysis, see Supplementary Appendix 1.

### Investigating bias as a result of loss to follow-up

Compared with those that did not respond to the follow-up questionnaire, a higher proportion of those that responded had no financial concerns at baseline (36.6% *v*. 44%) and a lower proportion met the screening criteria for depression at baseline (15.3% *v*. 12.1%). Responders had a higher median age than non-responders, and a higher proportion of responders (compared with non-responders) were from White ethnic groups (76.8% *v*. 67.6%).

## Discussion

In this analysis of a national cohort of 3521 HCWs, we found that those with financial concerns in December 2020 to March 2021 were more likely to develop symptoms of depression by June to October 2022 than those without such concerns. We also demonstrate that the proportion of HCWs concerned about their financial situation increased during the study period, with 43.8% reporting increased financial concerns and only 9% reporting decreased financial concerns. Predictors of developing financial concerns included younger age and working in a nursing or allied health professional role compared with a medical role.

Strengths of our study include the length of follow-up and large sample size. Ours is the first to use longitudinal data to examine the effect that financial concerns have on the risk of developing symptoms of depression in HCWs in the UK. Our work agrees with the existing literature. Sarfraz et al found an association between financial concerns and depression in a sample of HCWs in Pakistan.^10^ However, this study was limited by a small sample size, use of a non-standard depression screening tool and a cross-sectional design. In a USA cohort including (but not limited to) HCWs, financial concerns were associated with depressive symptoms.^25^ The ONS have determined that among British adults, prevalence of depression has increased between a pre-pandemic measure and early 2021 to a greater extent in those that indicated they were unable to afford an unexpected expense of £850 compared with those who could afford such an expense.^8^

Our study also has limitations. This questionnaire will likely suffer from response bias. Indeed our comparison of follow-up responders and non-responders indicates that those meeting the screening criteria for depression and those with financial concerns at baseline were less likely to respond to follow-up questionnaires (as has been described previously^26^), and this may have led to underestimates of the prevalence of depression and financial concerns. In comparison to the NHS workforce as a whole, our sample has a similar proportion of females and those from ethnic minority groups.^27,28^ The average age in our sample is higher than that of the NHS workforce,^29^ which could also have led to underestimates of the prevalence of financial concerns and depression. There are also some who may have left healthcare work as a result of financial concerns, depression or for other reasons. Inherent limitations exist in using a screening questionnaire to identify potential rates of depression. The PHQ-2 at a cut-off of ≥3 had a pooled sensitivity of 0.76 (95% CI 0.68–0.82) and a pooled specificity of 0.87 (95% CI 0.82–0.90), with substantial heterogeneity, in a 2016 meta-analysis.^30^ However, Löwe et al found that PHQ-2 change scores accurately reflected improved, unchanged and deteriorated depression outcomes in longitudinal data compared with the Structured Clinical Interview for DSM-IV (widely considered to be the gold standard for diagnosing depression in research).^31^ The low numbers of particular specific occupational groups within the cohort necessitated combining particular groups (e.g. combining midwives with nurses and pharmacists with allied health professionals) to minimise exclusions and maximise statistical power. This may have introduced heterogeneity in some levels of the occupation variable, and may have led to the grouping together of those with relatively high and relatively low wage brackets. Finally, as with all observational studies, the relationship between exposure and outcome may have been affected by unmeasured confounding.

Increasing financial concerns and the associated depression found among HCWs could have several important consequences for the UK healthcare workforce and its staff, including negative effects on staff recruitment, retention^32^ and sickness absence.^33^ This could, in turn, lead to adverse consequences for patient safety, the workload of the remaining staff and waiting lists. In accordance with the existing literature, we find that working in a nursing role represents a risk factor for depression.^1^ Concerningly, we also find that among the different HCW occupational groups in this study, nursing staff had the highest odds of financial concerns by Autumn 2022. These findings suggest that increasing financial concerns could further widen the gap in depression prevalence between nurses and their colleagues in other healthcare roles.

NHS pay has not kept pace with the retail/consumer price index or the rate of inflation over the past decade, and the gap is likely to increase substantially over the next year,^34^ particularly compared with private sector pay in what is a competitive skilled labour market. Therefore, the prevalence of financial concerns among HCWs is likely to rise. The Nuffield Trust estimates that the average NHS employee has lost 4.5% of pay (adjusted for inflation) from 2010–2011 to 2021–2022. They also predict that, for starting nurses and doctors, respective real-terms pay losses will double, with first year doctors predicted to be paid 12.6% less in 2022–2023 than in 2010–2011.^34^ Compounding this problem is the current ‘cost of living crisis’ in the UK, which the Office for Budget Responsibility has predicted will worsen in 2023–2024.^35^

This real-terms pay cut has often been cited as a primary contributor to strike action across the NHS workforce (alongside increasing demand and work pressures).^36–38^ Currently, the government has no plans to increase pay to a level that would rectify the real-terms losses. For comparison, the Trades Union Congress estimates that financial and insurance sector pay has almost doubled since 2008,^39^ suggesting that public sector workers may be particularly afflicted by financial concerns.

Mental health problems, including depression, are consistently the most frequently reported reason for sickness absence in the English NHS, accounting for over 496 400 full-time equivalent days lost and 24.9% of all sickness absence.^5^ Sickness absence has previously been estimated to cost the NHS over £1 billion per year.^40^ There is also a large deficit in the supply of skilled labour, which means that even the current rate of 5% sickness affects the performance of the service and the experience of staff and patients. Despite this, little attention is given to pay, its relationship to well-being or the experience of worker's mental health in workforce policy. The NHS England People Plan and People Promise,^41^ published before the COVID-19 pandemic, aimed to address workplace cultures, but had no remit over pay. Historically, pay has been recognised as a retention lever but these data suggest it is also a performance lever in terms of the availability of staff and therefore services, and should be considered in workforce policy decisions. In our study, nursing appears to be the workforce where financial concerns and depression were most associated. Nursing has large-scale retention issues, with almost 50 000 unfilled posts in England,^42^ and this study suggests a much closer relationship between financial reward and depression than a straightforward reward and recognition issue.

In summary, a large and increasing proportion of UK HCWs in this large nationwide study were concerned about their financial situation, and this was associated with developing symptoms of depression. Those in nursing roles were at particular risk of developing financial concerns and depression symptoms. The relationship between financial concerns and depression among HCWs has concerning implications for the future of a healthcare workforce already plagued by understaffing, particularly in light of the current pay disputes and cost of living crisis in the UK. Further research should examine whether the relationship between financial concerns and subsequent depression observed in this study contributes to workforce attrition.

## Data Availability

To access data or samples produced by the UK-REACH study, the Working Group representative must first submit a request to the Core Management Group by contacting the UK-REACH Project Manager in the first instance. For ancillary studies outside of the core deliverables, the Steering Committee will make final decisions once they have been approved by the Core Management Group. Decisions on granting the access to data/materials will be made within 8 weeks. Third-party requests from outside the project will require explicit approval of the Steering Committee once approved by the Core Management Group. Note that should there be significant numbers of requests to access data and/or samples, then a separate Data Access Committee will be convened to appraise requests in the first instance.

## Supplementary material

For supplementary material accompanying this paper visit http://doi.org/10.1192/bjo.2023.520. click here to view supplementary material
