# Supplementary material for: Investigating the impact of financial concerns on symptoms of depression in UK healthcare workers: data from the UK-REACH nationwide cohort study
Source: BJPsych Open. 2023 Jul 12;9(4):e124. doi: 10.1192/bjo.2023.520 (PMC10375869; doi:10.1192/bjo.2023.520)
Supplement: Supplementary file 1 [file S2056472423005203sup001.docx]

**Worsening financial concerns lead to symptoms of depression in UK healthcare workers: data from a nationwide cohort study (UK-REACH)**

Martin McBride^1*^, Christopher A. Martin^2,3*^, Lucy Teece^4^, Patricia Irizar^5^, Megan Batson^6^, Susie Lagrata^7^, Padmasayee Papineni^8^, Joshua Nazareth^2,3^, Daniel Pan^2,3,9^, Alison Leary^10^, Katherine Woolf^11^, Manish Pareek^2,3^, the UK-REACH Study Collaborative Group+

*Joint first authors / contributed equally

Corresponding author [manish.pareek@leicester.ac.uk](mailto:manish.pareek@leicester.ac.uk)

+Manish Pareek (Chief investigator), Laura Gray (University of Leicester), Laura Nellums (University of Nottingham), Anna L Guyatt (University of Leicester), Catherine John (University of Leicester), I Chris McManus (University College London), Katherine Woolf (University College London), Ibrahim Abubakar (University College London), Amit Gupta (Oxford University Hospitals), Keith R Abrams (University of York), Martin D Tobin (University of Leicester), Louise Wain (University of Leicester), Sue Carr (University Hospital Leicester), Edward Dove (University of Edinburgh), Kamlesh Khunti (University of Leicester), David Ford (University of Swansea), Robert Free (University of Leicester).

1. Department of Psychiatry, Leicester Partnership NHS Trust, Leicester UK
2. Department of Respiratory Sciences, University of Leicester, Leicester, UK
3. Department of Infection and HIV Medicine, University Hospitals of Leicester NHS Trust, Leicester, UK
4. Biostatistics Research Group, Department of Population Health Sciences, University of Leicester, Leicester, UK
5. Department of Sociology, School of Social Sciences, Faculty of Humanities, University of Manchester, Manchester, UK
6. Leicester Medical School, University of Leicester, Leicester UK
7. University College London Hospitals NHS Foundation Trust, London, UK
8. Department of Infectious Diseases, Ealing Hospital, London North West University Healthcare NHS Trust, London, UK
9. Li Ka Shing Institute for Health Information and Discovery, Oxford Big Data Institute, Oxford, UK
10. Department of Health and Social Care, London South Bank University, London, UK
11. University College London Medical School, London, UK

**Supplementary Figure 1. Recruitment and formation of the analysis sample**

*12,280 were recruited via email sent from regulators, 1018 from participating Trusts and 4593 by visiting the website directly or from social media advertising (see cohort profile for details^1^)

†This corresponds to 25.7% of those who started the first questionnaire, 19.6% of those who consented, and 13.2% of those who created a study profile.

We do not have data on the number of email invitations sent out by participating Trusts or on the number of people who viewed advertisements on social media so cannot determine what proportion of the total number of invitations received our analysis sample represents.

**Supplementary Table 1. Sensitivity analysis - Univariable and multivariable linear regression with an outcome of PHQ-2 score**

| **Variable** | **Unadjusted β (95% CI)** | **P value** | **Adjusted β**  **(95% CI)** | **P value** |
| --- | --- | --- | --- | --- |
| **Degree of concern about future financial situation**  Not at all  A little  Moderately  Quite a bit  Extremely | Ref  0.31 (0.22, 0.41)  0.67 (0.51, 0.82)  1.23 (1.00, 1.45)  1.47 (1.15, 1.78) | -  <0.001  <0.001  <0.001  <0.001 | Ref  0.12 (0.04, 0.20)  0.22 (0.08, 0.36)  0.50 (0.30, 0.70)  0.39 (0.10, 0.67) | -  0.005  0.002  <0.001  0.008 |
| **Age,** per decade increase | -0.17 (-0.21, -0.14) | <0.001 | -0.05 (-0.09, -0.02) | <0.001 |
| **Sex**  Male  Female | Ref  0.04 (-0.06, 0.15) | **-**  0.39 | Ref  0.04 (-0.06, 0.15) | **-**  0.004 |
| **Ethnicity**  White  Asian  Black  Mixed  Other | Ref  -0.05 (-0.17, 0.07)  -0.19 (-0.46, 0.09)  -0.02 (-0.25, 0.22)  0.03 (-0.36, 0.42) | -  0.44  0.19  0.87  0.89 | Ref  -0.03 (-0.15, 0.08)  -0.02 (-0.27, 0.22)  -0.06 (-0.26, 0.14)  -0.02(-0.36, 0.33) | -  0.55  0.87  0.57  0.91 |
| **Occupation**  Medical  Nursing (inc. HCA, NA, midwives)  Allied health professionals†  Dental  Admin/estates/other | Ref  0.35 (0.22, 0.48)  0.21 (0.10, 0.33)  0.35 (0.14, 0.56)  0.48 (0.27, 0.69) | -  <0.001  <0.001  0.001  <0.001 | Ref  0.12 (-0.01, 0.24)  0.06 (-0.04, 0.33)  0.11 (-0.07, 0.30)  0.26 (0.07, 0.45) | -  0.07  0.26  0.23  0.006 |
| **Index of Multiple Deprivation quintile**  1 (most deprived)  2  3  4  5 (least deprived) | 0.27 (0.08, 0.46)  0.05 (-0.11, 0.21)  Ref  -0.18 (-0.32, -0.05)  -0.23 (-0.36, -0.10) | 0.007  0.53  -  <0.001  <0.001 | 0.08 (-0.08, 0.25)  0.01 (-0.13, 0.15)  Ref  -0.08 (-0.23, 0.04)  -0.06 (-0.18, 0.05) | 0.35  0.87  -  0.18  0.27 |
| **PHQ-2 score at baseline**, per point increase in score | 0.48 (0.45, 0.51) | <0.001 | 0.44 (0.41, 0.47) | <0.001 |

†includes pharmacists, ambulance workers, healthcare scientists and those in optical roles

Supplementary Table 2 shows unadjusted and adjusted associations of baseline financial concerns and other demographic and occupational factors with PHQ-2 score (continuous variable, range 0 – 6) at follow up. Adjusted β coefficients are adjusted for all variables in the table. HCA – healthcare assistant, NA – nursing associate,

**Supplementary Text 1. Checking the parallel odds assumption after ordered logistic regression**

The parallel odds assumption for the ordered logistic regression model presented in Table 4 was checked using the Brant test, this was significant (p<0.001) implying that the proportional odds assumption was violated. To further investigate, we plotted odds ratios derived from logistic regression for each level of the outcome variable (i.e. not at all vs at least a little bit, not at all or a little bit vs at least moderately etc) and determined that the parallel odds assumption was likely violated by inclusion of baseline score. We tested this hypothesis by removing baseline score from the model and performing the Brant test again which returned a p value of 0.33.

Given the importance of adjusting for baseline score, but the lack of importance placed upon the relationship of this variable with the outcome and considering the problems that might be introduced by, for example, ignoring the ordering of the outcome variable and using multinomial logistic regression, we elected to continue with the planned analysis of using ordered logistic regression and adjusting for baseline score (accepting that the odds ratios for baseline score may not be accurate for all levels of the outcome variable).

**Supplementary Table 1. Comparison of baseline variables for follow-up questionnaire responders and non-responders**

| **Variable** | **Non-responders**  **N=9,085** | **Responders**  **N=3,521** | **P value** |
| --- | --- | --- | --- |
| **Degree of concern about future financial situation**  Not at all  A little  Moderately  Quite a bit  Extremely | 3,321 (36.6)  3,866 (42.6)  999 (11.0)  592 (6.5)  307 (3.4) | 1,550 (44.0)  1,451 (41.2)  312 (8.9)  140 (4.0)  68 (1.9) | <0.001 |
| **PHQ-2 score at baseline**  < 3  ≥ 3 | 7,695 (84.7)  1,390 (15.3) | 3,096 (87.9)  425 (12.1) | <0.001 |
| **Age,** med (IQR) | 43 (33 – 53) | 48 (37 – 56) | <0.001 |
| **Sex**  Male  Female | 2,108 (23.3)  6,953 (76.7) | 881 (25.1)  2,635 (75.0) | 0.03 |
| **Ethnicity**  White  Asian  Black  Mixed  Other | 6,036 (67.6)  1,847 (20.7)  436 (4.9)  394 (4.4)  214 (2.4) | 2,654 (76.8)  536 (15.5)  91 (2.6)  129 (3.7)  45 (1.3) | <0.001 |
| **Occupation**  Medical  Nursing (inc. HCA, NA, midwives)  Allied health professionals†  Dental  Admin/estates/other | 2,011 (23.1)  1,848 (21.2)  3,805 (43.7)  568 (6.5)  476 (5.5) | 819 (23.9)  756 (22.1)  1,471 (43.0)  196 (5.7)  183 (5.3) | 0.35 |
| **Index of Multiple Deprivation quintile**  1 (most deprived)  2  3  4  5 (least deprived) | 818 (10.2)  1,376 (17.1)  1,670 (20.8)  1,931 (24.0)  2,248 (28.0) | 279 (9.0)  479 (15.4)  624 (20.4)  771 (24.8)  941 (30.3) | 0.02 |

†includes pharmacists, ambulance workers, healthcare scientists and those in optical roles

All data are n(%) other than where specified. P values are from chi-squared tests for categorical variables and Wilcoxon rank-sum tests for age. In this sensitivity analysis we included only those who provided information on baseline depression score and baseline financial concerns (n = 12,606) Only those with complete data for the relevant variables are shown. Statistical tests were performed on those with complete data in the corresponding fields.

1. Bryant L, Free RC, Woolf K, et al. Cohort Profile: The United Kingdom Research study into Ethnicity and COVID-19 outcomes in Healthcare workers (UK-REACH). *Int J Epidemiol* 2022:dyac171. doi: 10.1093/ije/dyac171
